# Cellular and Molecular Adaptation of Bovine Granulosa Cells and Oocytes under Heat Stress

**DOI:** 10.3390/ani10010110

**Published:** 2020-01-09

**Authors:** Adnan Khan, Muhammad Zahoor Khan, Saqib Umer, Ibrar Muhammad Khan, Huitao Xu, Huabin Zhu, Yachun Wang

**Affiliations:** 1 Key Laboratory of Animal Genetics, Breeding, and Reproduction, MARA; National Engineering Laboratory for Animal Breeding, College of Animal Science and Technology, China Agricultural University, Beijing 100193, China; dr.adnan93@cau.edu.cn (A.K.); zahoorkhattak91@yahoo.com (M.Z.K.); 2 Embryo Biotechnology and Reproduction Laboratory, Institute of Animal Sciences, Chinese Academy of Agricultural Sciences, Beijing 100193, China; Saqibumar33@hotmail.com (S.U.); xuhuitao104@163.com (H.X.); Zhuhuabin@caas.cn (H.Z.); 3 Anhui Provincial Laboratory of Local Livestock and Poultry Genetical Resources Conservation and Breeding, College of Animal Science and Technology, Anhui Agricultural University, Hefei 230036, China; Ibrar.pesh@gmail.com

**Keywords:** heat stress, dairy cattle, granulosa cell, oocyte, cellular and molecular level

## Abstract

**Simple Summary:**

Heat stress can have large effects on most aspects of reproductive function in dairy cows. A hot environment can increase blood, rectal, and uterine temperatures, alter ovarian folliculogenesis, suppress fertility, oogenesis, and embryogenesis and ultimately reduce conception and pregnancy rates. Among the components of the female reproductive tract, the ovarian pool of follicles and their enclosed granulosa cells and oocytes are highly sensitive to hyperthermia. Many effects of elevated temperature on granulosa cells and developing oocytes involve increased production of reactive oxygen species, subsequently induce cellular apoptosis, and decrease the developmental ability of oocytes to be fertilized. Furthermore, heat stress-associated reproductive disorders are associated with altered progesterone and reduced estradiol production by ovarian follicles. The review mainly focuses on the follicle-enclosed granulosa cells and oocytes, provides new insights into the cellular and molecular adaptations of granulosa cells and oocyte under heat stress, depicts the role of the follicle microenvironment, and discusses some mechanisms that might underlie oocyte impairment. This study provides a possible way for the genetic adaptation to heat stress both for the regulation of body temperature and cellular resistance to elevated temperature.

**Abstract:**

Heat stress has long been recognized as a challenging issue that severely influences the reproductive functions of dairy cattle, disrupting oocyte development during fetal growth. These detrimental effects of heat stress are the result of either the hyperthermia associated with heat stress or the physiological adjustments made by the heat-stressed animal to regulate body temperature. In addition, elevated temperatures have been implicated in increasing the production of reactive oxygen species. Thus, understanding the impact of heat stress on reproductive functions, from a cellular to molecular level, might help in selecting heat-resilient dairy cattle and developing heat stress mitigation strategies. In the present paper, we have attempted to describe the changes in the reproductive system and function of dairy cattle in response to heat stress by reviewing the latest literature in this area. The review provides useful knowledge on the cellular and genetic basis of oocyte and granulosa cells in heat-stressed dairy cattle, which could be helpful for future research in this area.

## 1. Introduction

Heat stress induces infertility in dairy cows and represents a major source of economic loss to the livestock sector [1,2,3,4]. Mammals typically function at high body temperatures ranging from 35 to 39 °C, while the increase in body temperatures to levels higher than the surrounding environment is due to the high metabolic rate achieved by catabolism of energy resources. Body temperature is closely regulated by balancing heat production with heat loss to the environment via conduction, convection, radiation, and evaporation. The set point for body temperature regulation is not fixed, but may differ on a daily basis in some animals as part of the hibernation response or in response to changes in ambient temperature or other indications [5].

The mammalian ovarian follicle is composed of an oocyte enclosed by a layer of granulosa cells (GCs). During folliculogenesis, the oocyte experiences a chain of biological events (ovulation, fertilization, and embryo development) under the influence of signals and hormones produced by the hypothalamus–pituitary–gonadal (H–P–G) axis and the GC*s* [6,7,8,9]. Oocyte maturation and ovulation, proliferation, and differentiation of GCs is critical for normal follicular growth [10,11], and high environmental temperatures and humidity compromise fertility [12]. 

Heat stress (HS) is one of the environmental factors which has detrimental effects on ovarian function [13] and consequently decreases the developmental competence of oocytes to be fertilized and their further development into competent embryos [14]. The homeokinetic changes that regulate body temperature due to HS can impair reproductive function. Fertility impairment is a costly consequence of HS in hot and humid climates [15,16]. In a fertility study, cows were inseminated during the winter with semen collected either during the cold or hot season, and a lower pregnancy rate was observed for the semen collected during the hot season, signifying that oocyte quality—rather than that of spermatozoa—is the main reason for fertilization failure during the hot season. A study has shown that HS increases polyspermy during in vitro fertilization and reduces the conception rate by disrupting the antipolyspermy system in oocytes [17], indicating that HS during fertilization mainly affects the oocyte and its developmental competence. 

HS activates a dynamic gene expression system and adaptive biochemical responses [18]. These responses are a highly conserved cascade of protein activation and altered gene expression in response to a variety of stressors [19]. The gene expression component of this network is under heat shock transcription factor (HSF) regulation [20]. The central role that heat shock proteins (HSPs) have in cytoprotection during HS is demonstrated by the fact that HSP overexpression protects against hyperthermia, circulatory shock, and cerebral ischemia during heat stroke [21].

This review briefly discusses the impact of HS on ovarian function, focusing on its impairment of the follicle-enclosed cells, i.e., GCs and oocytes. New insights into heat-induced cellular and molecular alterations are presented. Two potential mechanisms (i.e., apoptosis and oxidative stress) by which HS disrupts ovarian cells (GCs) and oocytes are explored. Furthermore, the latest research findings and data on the impact of HS on GCs and oocytes are summarized. Due to the complexity of the problem and for the ease of readers, each section includes basic context and relevant knowledge related to the effects of HS in general, followed by specific impacts on ovarian physiology and oocyte function that are required for a clear understanding of dairy cattle reproduction.

## 2. Heat Stress Assessment

**Figure 1:**
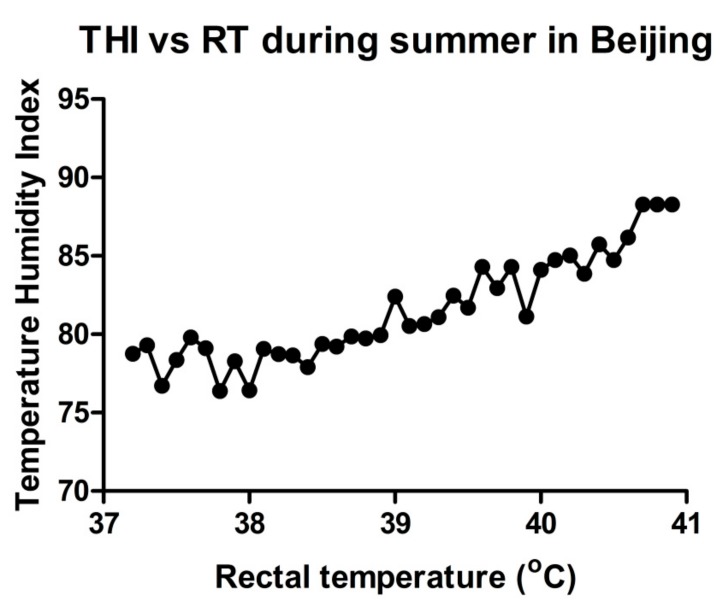
The temperature humidity index can affect rectal temperature (RT): Evaluation of change in RT with an increase in percent temperature humidity index (% THI).

The relationship between ambient temperature and rectal temperature (RT) has been studied extensively. In Florida, temperatures of 29.7 and 31.4 °C were correlated with an average RT of 39 °C (mild hyperthermia) and 39.5 °C (hyperthermia), respectively [22]. RTs greater than 39 °C represent a degree of HS that affects milk production and fertility [23,24]. Relative humidity (RH) also contributes to the intensity of HS in addition to the ambient temperature. Efforts were made to incorporate environmental factors into a single index, but the ratio of success was limited except for the temperature humidity index (THI) [25]. For instance, during the summer, we collected the data from many dairy farms in Beijing. THI was calculated based on the following formula [26]:

THI = (0.8 × T) + [(RH/100) × (T − 14.4)] + 46.4,(1)

where T: dry bulb temperature (°C); RH: relative humidity (%). The results showed that high environmental temperature and humidity, combined as THI, could affect RT. We found that in summer, the body temperature may rise to 41 °C (Figure 1). This study, to collect THI and RT data, was reviewed and approved by the Institutional Animal Care and Use Committee of China Agricultural University Beijing, China (permit number: DK996).

## 3. Impact of Heat Stress on Cow Reproduction

**Figure 2:**
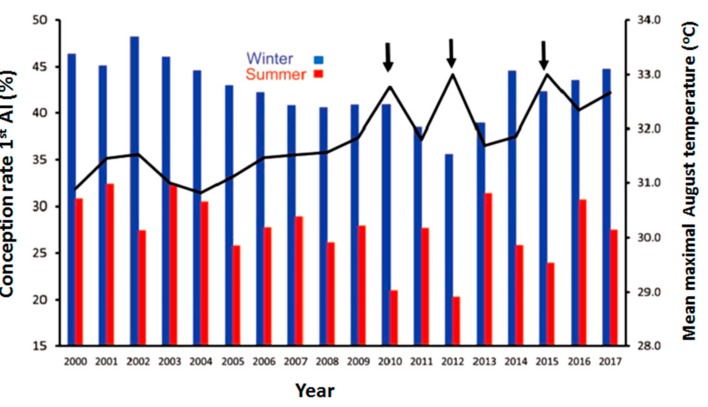
Conception rates of cows in dairy farms after the first insemination during the months of January–March (winter) or July–September (summer) of 2000–2017. The black curve represents the average environmental temperatures during August for each year. A noticeable decline in conception rates was observed during the intense conditions of summer in the years of 2010, 2012, and 2015.

The effects of HS on dairy cow reproductive efficiency are well known, including increased incidence of anestrous in dairy cows and days open through disruption of the pregnancy rate [27,28]. HS in mammals is known to alter follicular dynamics [29], steroidogenic ability [30], GC function [31], and oocyte maturation [32], and may lead to reduced reproductive efficiency. In a tropical climate like in India, the sensitivity of dairy cattle to HS increases with an increase in milk production [33], which might be due to increased metabolic heat production with increased production levels in dairy animals. Likewise, HS results in maternal hyperthermia by increasing RT, which is responsible for impaired reproduction [34]. According to one large-scale retrospective study, the observed average rates of pregnancy to first artificial insemination (AI) and anestrous rates were respectively 44% and 27% for the cool period and 1.2% and 12.9% for warm [35]. This is seen in Figure 2, which demonstrates the depressive effect of summer heat on the conception rate of lactating cows artificially inseminated in the summer months over the last 18 years, as low as 27.7% compared to 42.6% during the cool winter months. Moreover, the slightly more severe conditions during the summers of 2010, 2012, and 2015, about 1.5 °C above average summer air temperatures, further decreased conception by an additional 5% units (Figure 2) [36]. Therefore, the deteriorating effects of HS on reproductive efficiency during hot weather are vital in terms of the economic damage in addition to losses due to lower milk production. 

### Hormonal Regulation and Estrous Behavior

Since the release of gonadotrophin-releasing hormone (GnRH) from the hypothalamus and the gonadotrophins, luteinizing hormone (LH), and follicle-stimulating hormone (FSH) from the anterior pituitary gland are the main factors which control ovarian function, some researchers have studied the impact of HS on the secretion of these hormones [37,38,39,40]. The observed effects of on LH concentrations in peripheral blood are inconsistent. Some studies reported unchanged concentrations [41], while others reported lower levels of steroids under gonadotropin stimulation following HS [40]. A decrease in LH pulse amplitude [42] and LH pulse frequency [43] has been reported with respect to the pattern of LH secretion in heat-stressed cows. Similarly, the effect of HS on the preovulatory LH surge is also controversial; a reduction in the endogenous LH surge by HS has been reported in heifers but not in cows. The reasons for these inconsistencies are unclear. These variations are suggested to be linked with preovulatory estradiol levels because the frequency of tonic LH pulses and preovulatory plasma LH surges caused by GnRH were decreased in cows with low plasma concentrations of estradiol (E2), but not in cows with high plasma concentrations of E2 [42]. The development of substandard corpus luteum (CL) with lower progesterone secretion might be due to the low LH surge. Additionally, impaired GnRH secretion in the summer can reduce cow fertility. Administration of GnRH for treating cystic ovaries and inducing ovulation appears to be a suitable approach for correcting the situation because during the estrous cycle, GnRH induces LH release, which makes the dominant ovarian follicles ovulate and enhances the conception rate in heat-stressed cows [44]. Since most of the studies found that HS is responsible for low levels of LH, we are led to infer that in summer, the dominant follicle grows in a low LH environment, resulting in reduced estradiol secretion from the dominant follicle, leading to poor expression of estrus and, hence, reduced fertility. Furthermore, during summer, plasma inhibin concentrations are lower in both heat-stressed cows [45] and cyclic buffaloes in India [46], possibly causing reduced folliculogenesis as a significant proportion of plasma inhibin was extracted from small- and medium-sized follicles.

**Figure 3:**
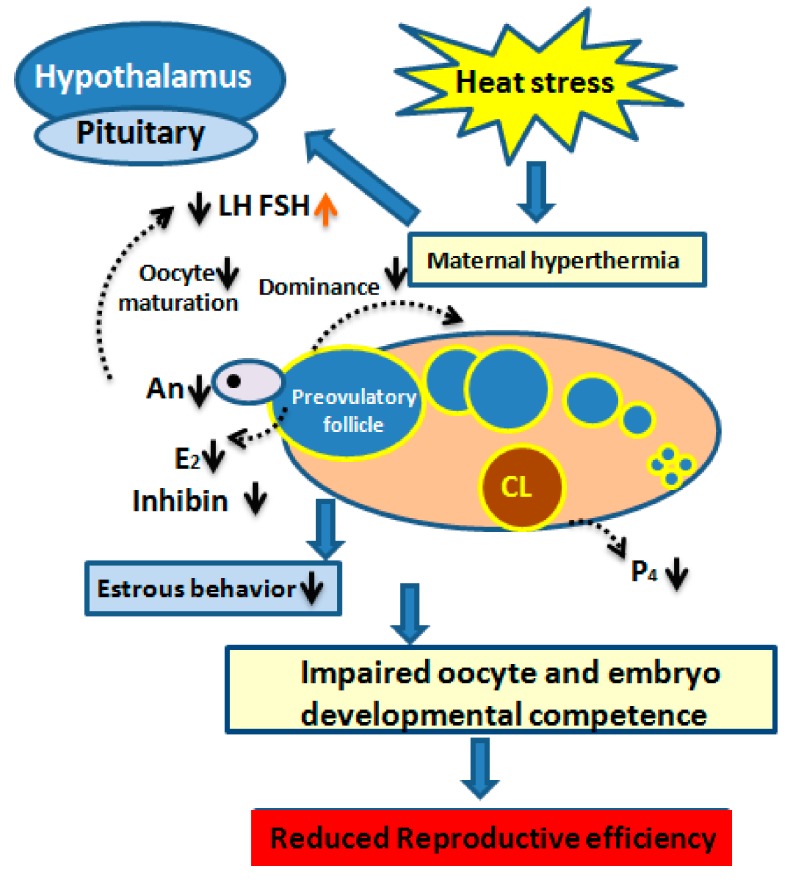
Diagram elucidating the interaction between the impact of seasonal heat stress and the hypothalamus–pituitary–ovarian axis and its mechanism of affecting dairy cow fertility. The lower luteinizing hormone (LH) surge is related to a decreased secretion of follicular estradiol (E2). Lower levels of androstenedione (An) and E2 levels are responsible for decreasing the dominance of preovulatory follicle and is related to poor estrous behavior. Likewise, impaired concentrations of follicle-stimulating hormone (FSH) and inhibin are related to the increased number of medium-sized follicles. Impaired competence of maturing oocyte and developing embryo is due to the disturbed nuclear and cytoplasmic maturation. Reduced reproductive efficiency in dairy cows is the ultimate outcome of heat stress.

Mammalian estrus also acts as a behavioral indicator, confirming if the female is bred near ovulation time. The observed core outcome in heat-stressed cows that up to 80% of estruses failed to be detected [47] due to a seasonal effect on estrous behavior [48]. Long-term exposure to a high ambient temperature compromised the conception rate by shortening the duration and reducing the intensity of estrus signs [49,50]. Moreover, hot weather during summer causes ovulation without showing signs of estrous [51]. Most likely, the lower level of blood E2 due to the disrupted steroidogenic potential of heat-stressed GCs is considered as the main cause of impaired heat detection [52]. Nonetheless, the cumulative pattern of impaired reproductive efficiency induced by heat stress [53] and is shown in Figure 3. 

## 4. Impact of Heat Stress on Granulosa Cell Function

GCs are somatic cells and are considered the most important ovarian cell type that surround, support, and nurture the developing oocyte physically and provide a suitable microenvironment for its maturation [54]. In the early phase of the primordial follicle, cells are incapable of producing steroidal hormones [54,55]. However, FSH stimulates GCs in the developmental stages before ovulation to convert androgens to estradiol via cytochrome P450 aromatase. After ovulation, GCs turn into progesterone-producing granulosa lutein cells. Progesterone (P4) plays a key role in maintaining pregnancy. One of the key roles of GCs is in the synthesis of two main reproductive steroid hormones in the ovary, i.e., E2 and P4 [56,57]. Estradiol plays an important role in the development of ovarian follicles, oocyte maturation, and endometrial proliferation. GCs have specific receptors for the gonadotropins FSH and LH [54], insulin-like growth factor (IGF) [58], and anti-Müllerian hormone (AMH) [59,60]. Any disturbance of GC quality and their proliferation capability may also have indirect effects on the development of the follicle and may disrupt oocyte maturation and, consequently, result in impaired embryo development with unsatisfactory pregnancy outcome [61]. 

HS is one of the compromising factors that affect the normal physiological functions of GCs by increasing intracellular accumulation of ROS, inducing apoptosis, and reducing the synthesis of E2 and P4 [52,62,63]. HS led to the induction of HSP genes in GCs [64,65,66]. Likewise, HSP induction was also reported in various cell/tissue types, such as leukocytes/lymphocytes [67,68,69], bovine endometrial tissue, bovine conceptuses [70,71], bovine MECs [72], and buffalo lymphocytes [73] due to HS. Moreover, it has been reported that HS causes an increase in HSPs in virtually all vertebrates, including mice [74,75], domestic goats [76], humans [77,78], juvenile hamadryas baboons [79], common carp [80], domestic chickens [81,82,83], and domestic turkey [84], thus supporting the idea that HSP70 can act as a reliable thermal stress biomarker. Upregulation of apoptotic genes (*BCL2, BAX, CASP3*) under HS could lead to a disruption of the potential of mitochondrion transmembrane, resulting in the release of cytochrome c and cellular apoptosis [85]. However, the mitogen-activated protein kinase-mediated induction of HSP70 at high temperatures could play a crucial role in inhibiting Caspase-3 and *BAX* activation [86,87]. Therefore, we suggest that the induction of HSP70 might play a role in reducing apoptosis of GCs induced by HS. 

HS results in intracellular ROS accumulation, causing oxidative stress [88] and apoptosis [89], which subsequently lead to a decline in fertility [31,90]. In response, the activation of fork head box O3 (FOXO3) and kelch-like ECH associated protein 1 (KEAP1) under HS protects cells from oxidative stress by upregulating antioxidant enzymes superoxide dismutase 2 (SOD2) and catalase (CAT) [60,91,92]. In *Saccharomyces cerevisiae* and quail, genes from the glutathione peroxidase family were also shown to be induced under HS [93,94]. Based on these facts, it is reasonable to suggest that the upregulation expression of SOD2 and CAT may inhibit ROS biosynthesis through the regulation of KEAP1 and FOXO3 in ovarian GCs.

**Figure 4:**
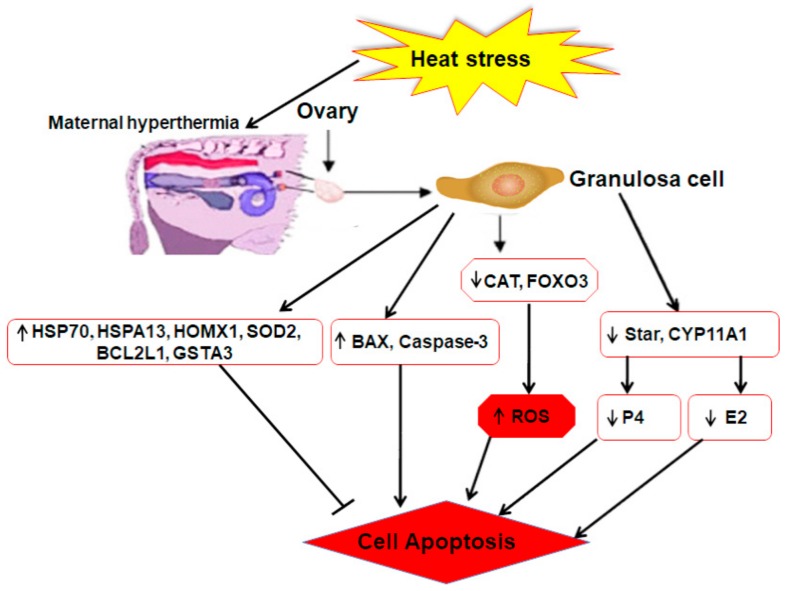
Mechanisms of regulating heat stress response related to follicular function within bovine ovary. Upregulated genes caspase-3, superoxide dismutase (SOD), *BCL-2*, and *BAX*, and heat shock proteins (HSPs) (HSP70, HSPA13, HMOX1) are involved in the regulating mechanism of bovine *GCs* via induced or inhibited cell apoptosis. Under heat stress, downregulated genes *CAT* and fork head box O3 (FOXO3) were involved in the production of reactive oxygen species (ROS). Likewise, downregulation of steroidogenic acute regulatory protein (STAR) and cytochrome P450, family 11, subfamily A, polypeptide 1 (*CYP11A1*) were involved in the secretion of E2 and progesterone (P4). Moreover, the decline of E2 and the enhancement of ROS might, in turn, enhance the possibility of *GC* apoptosis and follicle function.

Moreover, the regulation of genes related to steroidogenesis (E2 and P4), i.e., steroidogenic acute regulatory protein (STAR) and cytochrome P450, family 11, subfamily A, polypeptide 1 (*CYP11A1*) was affected by heat shock. Positive regulation of P450 aromatase family genes, such as *CYP11A1*, in the ovarian follicle promotes estrogen biosynthesis [95]. In addition, progesterone is also one of the fundamental steroid hormones for bovine estrous cycle regulation, and its biosynthesis is attributed to the increased expression of STAR and *CYP11A1* [96,97,98]. Previously, it was reported that under HS, the mRNA expression of *CYP11A1* and STAR decreased, but the P4 level had no significant (*p* < 0.05) difference between the control and heat treatment group [62]. Some studies reported an oversecretion of ovarian hormones in porcine ovarian GCs under high temperature [99]. Shown in Figure 4 is a brief overview of the mechanisms of regulating HS response which are related to GC function within the bovine ovary.

## 5. Impact of Heat Stress on Ovarian Pool of Follicles and Oocyte Quality

Folliculogenesis is the formation of a mature and/or Graafian follicle from a pool of primordial, non-growing follicles, and it is a long and highly dynamic process that occurs during the follicular phase of the estrous cycle of the female. A primordial follicle consists of an oocyte enclosed by a layer of flattened GCs. At birth, the ovary of the newborn heifer may approximately contain a total of 150,000 primordial follicles. This number decreases with the age of the cow, and after 15–20 years, it declines to 1000 follicles. Follicular development in cattle [100,101] occurs in a wave-like pattern. The emergence of each follicular wave is stimulated by an FSH surge [102]. After emergence, follicles enter a common-growth phase, and the FSH surge begins to decline, reaching the lowest levels near the time of deviation [103]. This decline in FSH has been identified as a key component of the selection process because experimental administration of FSH during the common-growth phase of the wave in cattle [104] prevented deviation and allowed the development of multiple dominant follicles. A transient increase in circulating LH surrounding deviation has been reported in cattle [105,106]. Additionally, the expression of granulosa LH receptors has been reported to increase near deviation in the future dominant follicle [107,108]. The acquisition of LH receptors in the GCs of future dominant follicles might allow the LH to transiently increase and have a functional effect in the selection process. In this regard, LH is known to stimulate and increase estradiol concentrations in follicular fluid, which is involved in the continuing depression of FSH to concentrations lower than those required by the smaller follicles, thus facilitating the establishment of dominance [109]. The complete phase of bovine follicular development takes approximately 180 days [110]. Follicles at a primordial stage are considered to be insensitive to heat because no data are available that shows their sensitivity. Most of the studies focus on the impact of HS on the ovarian pool of follicles. The sensitivity and tolerance of the developing follicles and their enclosed oocytes to heat stress [53]. Follicular development from the primordial to the preovulatory stage involves three phases [111,112]. The first is the gonadotropin-independent phase that consists of the primary and secondary follicles, while the gonadotropin-dependent phase comprises early antral to preovulatory follicles. The sensitivity of follicles and developing oocytes increases from the primordial stage to the preovulatory stage [113,114]. 

Oocyte development is extremely sensitive to high temperatures. During follicular growth, the developmental potential of the oocyte is gained in a stepwise pattern and, thus, the disruption in the follicular microenvironment and their function induced by heat stress may result in impaired oocyte competency. During the summer, oocytes collected from Holstein cows were delayed in the first two embryonic divisions [52,115]. A period of two to three consecutive estrous cycles may be needed for the recovery of an oocyte from summer heat damage and, further, to regain competency in the following autumn [116], suggesting a long-term consequence of HS on the ovarian pool of oocytes. This might be the reason that even during autumn (i.e., when there is no environmental thermal stress), the fertility of cows is compromised. It is notable that maternal hyperthermia results in the impairment of only a subpopulation of the ovarian follicles, rather than the entire follicular pool, as evidenced by the spontaneous restoration of the oocyte competence and pregnancy rate in the autumn and subsequent winter. Failure of oocyte maturation under HS happens in many ways. It disturbs the biosynthesis of steroid hormones, such as LH, that are involved in the regulating mechanisms of oocyte maturation [52]. In addition, it also induces oxidative stress because HS enhances the production of ROS and lowers the level of glutathione and as was found in the heat-stressed oocytes and embryos of mouse [16,117,118]. Oxidative stress is one of the most common factors that compromises normal cellular physiological functions, destroys the intracellular structure, and eventually leads to cell death. Thus, it harms oocyte maturation in both in vivo and in vitro environments. It was previously stated that HS (42 °C) encourages apoptosis in surrounding cumulus cells and decreases the oocyte maturation rates during the germinal vesicle breakdown stage [119]. Furthermore, a significantly increased ratio of ROS/GSH was reported in the heat-stressed oocyte [118]. Similarly, HS can block the nuclear maturation of oocytes, leading to a decrease in polar body rate. For example, upon exposure to a temperature of 41 °C, the maturation rate of bovine oocytes was significantly reduced [120]. Furthermore, HS can also disrupt the cytoskeletal structure of oocytes [121]. On the other hand, HS highly regulates the gene expression of *HSP70*, the apoptotic gene *caspase-3*, and other antioxidant-related genes (*SOD1*, *CAT*, and *CPX4*) in cumulus oophorus complex (COC). These changes in gene regulation imitate the self-defense mechanisms of COCs under HS. HSP70 is a multi-effect factor, maintaining stability of the intracellular environment and inhibiting cell apoptosis [122]. The expression of HSP70 can also protect cells from apoptosis by regulating *Caspase-3* and cytochrome c [123]. Therefore, it could be inferred that a high level of HSP70 helps oocyte survive from HS through upregulation of the *SPKH1, BCL-2, SOD1, CAT,* and *CPX4* and downregulation of *p53*.

## 6. Conclusions

Heat stress affects the reproductive efficiency of dairy cattle in a variety of ways. Understanding of the cellular and molecular responses of oocyte and GCs in response to HS will be helpful in developing strategies for heat resistance in dairy cattle. The genes regulated by heat stress are potential heat resistance markers that can be targeted in dairy cattle.
